# Neural evidence that disengaging memory retrieval is modulated by stimulus valence and rumination

**DOI:** 10.1038/s41598-020-64404-7

**Published:** 2020-05-05

**Authors:** Jiangyi Xia, Lisa H. Evans

**Affiliations:** 1 Center for Mind and Brain and Neurology Department, University of California, Davis, 267 Cousteau Place, Davis, 95618 California USA; 2 Cardiff University Brain Research Imaging Centre (CUBRIC), School of Psychology, Cardiff University, Cardiff, CF24 4HQ, Wales, UK

**Keywords:** Cognitive control, Cognitive control, Human behaviour, Human behaviour

## Abstract

To remember information from our personal past we need to be in a cognitive state where we treat stimuli as cues for memory retrieval. In this study we considered whether participants could exert control and disengage from a memory state when it was no longer required for the task at hand. In particular, we examined whether this ability was affected by the valence of the stimuli and participant’s rumination scores. After a study phase participants completed test blocks where the task switched every two trials between a memory task (retrieving information from the study phase) and a perceptual task. Even though there was no episodic memory requirement in the perceptual task, a well-established event-related potential (ERP) index of memory retrieval was present for both trials when the stimuli were negative valenced pictures but not for neutral pictures. Furthermore, there was a positive correlation between the magnitude of this ERP memory index in the perceptual task and rumination scores but only for neutral stimuli and not negative. Thus, in this study participants generally had difficultly suppressing memory retrieval when negative stimuli were presented. However, for neutral stimuli only ruminators were more susceptible to memory intrusions.

## Introduction

Many of the places, people and objects that we encounter in everyday life have associations with our personal past. However, even though our environments can contain numerous memory cues we do not typically spend our time constantly reminiscing about past events. Tulving^1^ argued that individuals need to be in a cognitive state which is focused on their personal past to recover this information. A few studies have examined the initiation of memory states^2,3^ and more recently the beneficial effects that they have on episodic memory retrieval^4–6^. However, in this paper we consider the opposite: the extent to which people can disengage from a memory state and the extent of unwanted retrieval when they move on to a different task. These studies offer a closer parallel to how memory operates in the real-world; where we typically switch between cognitive processes, some requiring memory, others not, rather than continuously recalling past events. At issue in this study is whether we can curtail remembering when it is no longer required for the task at hand and how this might be affected by the valence of the stimuli and individual differences in ruminative tendencies.

There is a rich literature on active suppression of unwanted memories, where participants are explicitly told to stop a particular memory coming to mind. The most prevalent and well-researched paradigm is the think/no-think (TNT) task^7^. Initially participants must learn several word pairs. In the second stage, participants are given the first word of the pair with the instruction to retrieve (think condition) or to prevent retrieval (no-think condition) of the associated word. In the no-think condition the participant must overcome the strong tendency to recall the second word in the pair. In the final test phase participants are given the first word of the pair and need to remember the word that was paired with it. Numerous studies have demonstrated that recall of no-think word pairs is significantly poorer than word pairs which were i) in the think condition and, ii) a baseline condition (reviewed by Anderson & Levy^8^ although see^9^ for a failure to find memory suppression in a direct replication). This latter finding suggests participants can engage suppression mechanisms that allows them to prevent access to unwanted memories and helps them to forget. Retrieval suppression enhances activity in brain regions associated with active control, such as lateral prefrontal cortex and anterior cingulate cortex, and decreases neural activity in regions linked to memory recollection, such as the hippocampus^10^.

One question which has been examined is whether the valence of the stimuli makes a difference to people’s ability to suppress memories. Some researchers have argued that due to the distressing nature of unpleasant memories people might be more motivated and able to suppress them^11,12^. Furthermore, evidence suggests that greater cognitive control can be exerted over stronger memory representations^13^, which is likely to be the case for emotional compared to non-emotional stimuli. However, there is also an opposing view that negative memories will be less amenable to suppression. We know that generally events which are associated with strong emotions are better remembered than those that do not have an emotional component^14–18^. This emotional enhancement effect has been found using words, sentences, pictures and narrated slide shows (see reviews by^19,20^). Moreover, using both subjective and objective measures it has been found that participants’ memories for unpleasant events are extremely vivid and contain more contextual details than neutral events^16,18,21,22^. Using the TNT paradigm a range of results have been found: some studies have found that suppression for negative stimuli is enhanced^23,24^, decreased^25–27^ or no different^28,29^ from neutral stimuli.

In studies using the TNT paradigm there is an overt instruction for participants to prevent a certain memory coming to mind and this is a central aspect of their task. In our work we wanted to consider a different circumstance: the extent to which individuals would continue to engage episodic memory processing when the subsequent task had no requirement to do so. This would provide complementary findings to those obtained using the TNT paradigm about the circumstances under which individuals can exert memory control to prevent memory retrieval when it is not required for the task at hand. One of the challenges in addressing this research question is knowing if participants are retrieving information from memory when they are completing another task. Critically, using electrophysiological data this can be achieved because there is a distinct event-related potential (ERP) index of recollection (the recovery of contextual details surrounding an event) called the left-parietal ERP old/new effect. This effect is characterised by relatively greater positivity for correctly identified old items compared to new items, and it is maximal at left-parietal scalp locations from around 500 ms. The evidence linking the left-parietal ERP old/new effect to recollection is substantial (for reviews see^30–32^) and it has been used in numerous experiments as a proxy for recollection in the absence of direct behavioural data e.g.^33–37^.

Previously we have used the left-parietal ERP old/new effect to gain insight into whether participants continue to retrieve episodic information when there is no requirement to do so and the behavioural consequences^38^. In the study phase participants indicated if a neutral word appeared to the left of fixation or right. At test these words were randomly intermixed with new ones and participants were cued to switch between completing two different tasks: one requiring memory of study item location and the other had no episodic memory component. These tasks alternated every two trials. As would be anticipated the left-parietal ERP old/new effect was present in the episodic task. Interestingly, this index was also observed in the non-episodic memory task for the first trial, i.e. after participants had just switched from completing the memory task (known as the switch trial) and was greatly diminished on the second trial (the stay trial). This result is consistent with the view that memory processes engaged on the preceding task carried over and remained active on at least the first trial of the subsequent task.

The main aim of the current study is to determine the effects of emotional stimuli on the ability to curtail remembering when it is no longer required for the current task and is unwanted. The design will closely mirror our earlier study^38^, except that negative and neutrally valenced pictures will be used. In the previous study, using neutral words, a left-parietal ERP old/new effect was statistically significant on the switch trials only and so we predicted the same pattern in this study using neutral pictures. It is unclear what would be predicted with negative stimuli from the TNT literature as the results are inconsistent. However, given the emotional enhancement effect we hypothesised that participants would be less able to curtail remembering when the stimuli are negative. Thus, here we anticipated that the left-parietal ERP old/new effect would be more persistent to negative stimuli in the non-episodic task and present on both switch and stay trials.

A more exploratory aim was to consider whether there would be any differences in the extent to which participants continued to recover memory information in the non-memory task as a function of rumination. This has been described as repetitive processing of negative thoughts or experiences, which are unwanted and cause the individual distress (see^39^ for a detailed consideration of the concept). Rumination is thought to play a role in various kinds of psychopathology, such as depression, anxiety as well as the maintenance of wellbeing^40^. Some researchers have suggested that a general problem with disengaging attention from distracting or irrelevant information is what predisposes an individual towards rumination in the first place^41^. Using the TNT paradigm with both neutral and emotional stimuli it has been found that those high in rumination have better memory in the final test for items which they had tried to prevent retrieval for previously^42,43^. As these findings indicate that those high in rumination have poor control over memory we predicted that there would be a positive correlation between rumination scores and the magnitude of the left-parietal ERP old/new effect in the non-episodic condition with both negative and neutral stimuli using our task.

## Materials and Methods

### Participants

Forty-three healthy volunteers took part in the experiment after giving informed consent in exchange for course credits or payment. All participants reported they were right-handed native English speakers who had normal or corrected-to-normal vision with no self-reported diagnosis of a psychiatric or neurological condition. Eleven participants were excluded from the study due to a failure to contribute at least 16 artefact-free trials to the conditions of interest. Of the 32 participants included, 31 were females and all were aged between 18 and 24 years (mean age: 20). Ethical approval for the study was granted by Cardiff University’s School of Psychology Ethics Committee and all procedures were carried out in accordance with the British Psychological Society’s guidance.

Evans *et al*.^38^ reported an effect size (Cohen’s d_z_) of 0.66 on switch trials at the P3 electrode size for the left-parietal ERP old/new effect in a non-memory task. Assuming a power level of 0.80 and an alpha of 0.05 this leads to an estimated average sample size of 21 to obtain similar effects. A sample size of 32 participants was predetermined based upon this calculation and counterbalancing considerations.

### Stimuli and design

A total of 572 pictures were taken from the International Affective Picture System (IAPS^44^), half negative and half neutral. From this pool of pictures 240 negative and 240 neutral pictures were selected as the test stimuli. According to the normative rating data, on a scale from 1 to 9, the mean valence ratings were 2.78 (range: 1.31–3.92) for the negative pictures and 5.17 (range: 4.13–6.21) for the neutral pictures selected, which were significantly different (*t*(478) = 40.94, *p* < 0.001). Mean arousal ratings were 5.60 (range: 3.52–7.53) for negative pictures and 3.87 (range: 1.72–6.97) for neutral pictures, which also differed significantly (*t*(478) = 19.45, *p* < 0.001).

Twelve pictures were used to create practice lists. Each participant completed 20 study–test cycles, half negative and half neutral. The study-test cycles were blocked by valence and counterbalanced i.e. all the negative items were processed before the neutral items, or vice versa. Within each cycle, 12 pictures were shown at study and again at test together with 12 new pictures. At study, half of the pictures were presented to the left of central fixation and the other half to the right. The order of left/right presentation location was randomised with the constraint that no more than 3 trials in a row could be at the same location. At test, each picture was preceded by a cue (X or O) which indicated which task participants should complete on the subsequent picture. The cue-task correspondence was counterbalanced across participants. In addition, for each valence set the old/new status, episodic/location task assignment, and left/right presentation location were counterbalanced across participants. At test all pictures were presented above, at, or below the central fixation, with an equal number at each location.

For each valence category, an additional 40 pictures (4 for each study-test cycle) were selected as filler trials to be inserted into the test phase trial sequence to render it less predictable i.e. there could occasionally be three trials of the same task or only one, as well as the more usual two trials of the same task. An example sequence from one test phase could be (filler trials underlined): XXOOXXXOOXXOXXOOXXOOXOOOXXOO. The filler trials were not included in the analyses. In the task switching literature two types of tasks are prevalent: the alternating-runs paradigm in which the task alternates, usually every 2 trials, which is constant and predictable; and the task-cueing paradigm where a cue appears either with or just before a stimulus and the sequence is unpredictable, see^45^ for more details. Our task contained elements of each of these types of paradigms, using a design which has been used in previously published work e.g.^5,46^.

Pictures were shown on a computer screen on a grey background, each subtended a visual angle of 5.3° horizontally and 4.0° vertically when presented at the centre of the screen. During the study phase, each picture appeared at an additional ±1.14° to the left or right of the central location. During the test phase, pictures appeared either in the centre of the screen, or an additional ±1.07° above or below.

The Ruminative Responses Scale^47^(taken from the Responses Styles Questionnaire^48^) was used to assess participant’s ruminative tendencies. This scale has 22 items which participants are asked to rate according to what they generally do, on a 4-point scale: 1 = almost never, 2 = sometimes, 3 = often, and 4 = almost always. Answers are summed, to give a minimum score of 22 and a maximum possible score of 88. The scale has very high internal validity, with Cronbach’s alphas of around 0.9^47^ and many studies have demonstrated its predictive validity^48^. The mean score obtained in this study was 41.53 (SD = 13.47), with a range of 24–72.

### Procedure

Participants completed the questionnaire prior to being set up for EEG. At the start of the task there was a practice session with 12 pictures, which participants could repeat until they were ready to move on to the experiment proper. In the study phase participants were asked to memorise the location of each picture and press a button to indicate whether it was left or right using the index and middle fingers of one hand. A trial began with a fixation asterisk (900 ms), a blank (100 ms), the picture (2000 ms), followed by another blank screen (500 ms).

At test each test picture was preceded by one of two cues, indicating the type of task participants had to perform: the episodic location task and the perceptual location task. The episodic location task cue directed participants to recollect whether the picture appeared on the left or right side of the screen at study or whether it was a new picture (Left/Right/New), see studies^2,4–6^ for others who have used a source decision and new as test response options. Participants respond with the same fingers of the same hand as at study for left and right, and the index finger of the other hand for new; the left and right hands were counterbalanced across participants for old and new responses. In the perceptual location task participants were required to indicate if the picture was presented at the top, middle or bottom of the screen by button presses using the same three fingers as for left, right, and new respectively.

**Figure 1:**
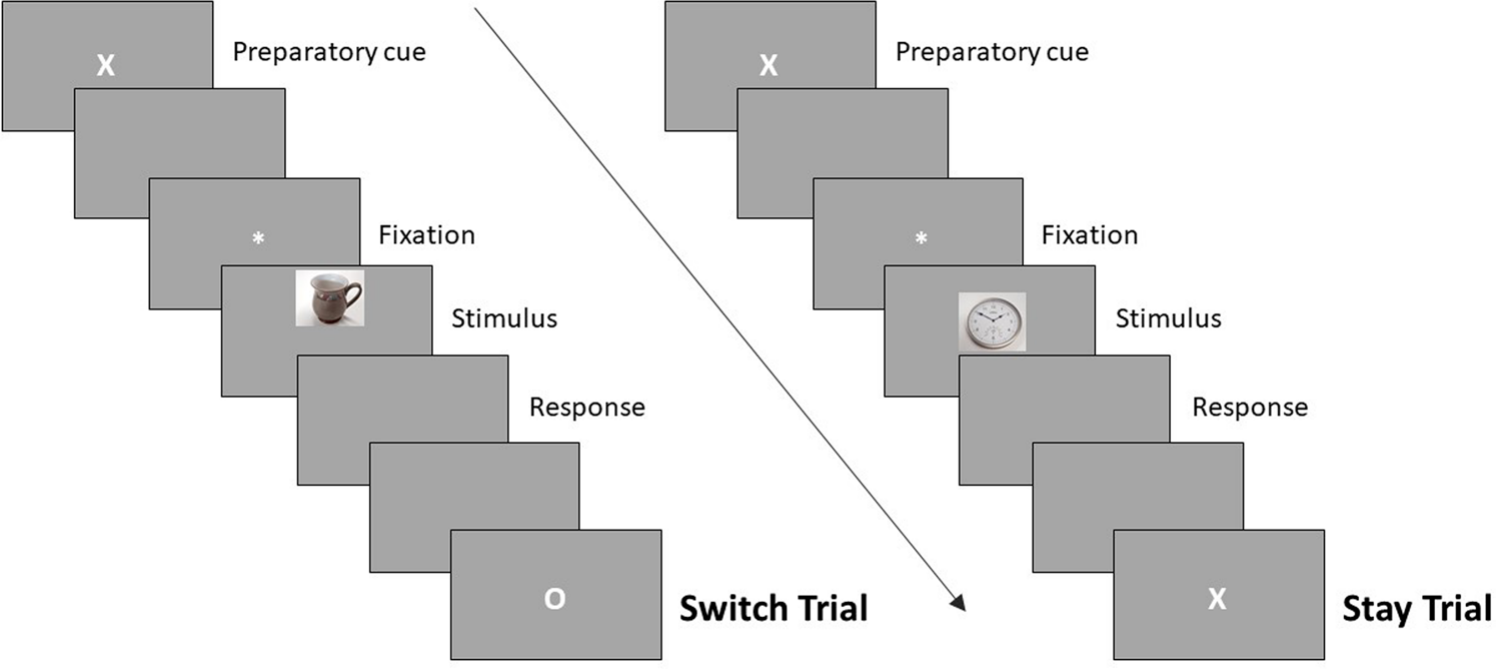
A schematic of the trial sequence in the test phase illustrating a switch trial, where the task changes from one trial to the next, and a stay trial, where the task remains the same from one trial to the next. X and O denote the task the participant is asked to complete on the up-and-coming trial (Episodic or Perceptual). Timings and example task sequence can be found in the main text. Pictures are examples and not from the IAPS^44^.

The preparatory cues were presented for 500 ms followed by a blank screen (100 ms), an asterisk (1700ms), the picture (500 ms), and the screen was then blanked until a response was made. After a response the screen remained blank for a further 700 ms before the onset of the next trial. All pictures at test were presented at top, middle, or bottom screen locations, regardless of task. See Fig. 1 for an overview of the procedure at test. Participants were instructed to pay attention to the preparatory cue in order to identify the task requirements, to balance speed and accuracy equally and to fixate eye-gaze centrally throughout the study and test phases. Trials on which responses were faster than 300 ms or slower than 4000 ms were counted as errors and excluded from the behavioural analyses (0.39% of the trials in the categories of interest), the same as the criteria adopted in^49^.

### Electroencephalogram (EEG) acquisition

EEG was recorded during the test phases using a Biosemi ActiveTwo amplifier from 32 scalp locations based on the international 10–20 system^50^. These included midline (Fz, Cz, Pz, Oz), fronto-polar (Fp1/Fp2), frontal (F7/F8, F5/F6, F3/F4, F1/F2), central (C7/C8, C5/C6, C3/C4, C1/C2), posterior (P7/P8, P5/P6, P3/P4, C1/C2), and occipital (O1/O2) sites. Additional electrodes were placed on the left and right mastoids and vertical and horizontal eye movements were recorded from above and below the right eye and from the outer canthi. EEG data were recorded at 2048 Hz and were acquired referenced to linked electrodes located midway between POz and PO3/PO4 respectively and were re-referenced off-line to the average of the signal at the two mastoids. EEG data were filtered off-line (0.03–40 Hz) and downsampled to 167 Hz for retrieval items. Trials containing large EOG artifacts were rejected, as were trials containing analog-to-digital saturation or baseline drift exceeding ±80 μV. Other EOG blink artifacts were corrected using a linear regression estimate^51^. The researcher was blind to trial identity when processing EEG data. Procedures for the rejection of trials were set a priori and were based on standards we have adopted in previous work^4,35,38^. The total epoch length was 1536 ms with a 102 ms prestimulus baseline.

## Results

The term source hit is used for correct location judgements to old pictures in the episodic task and perceptual hits for accurate responses to the location of words in the perceptual task. Correctly classified new test items in the episodic task are referred to as correct rejections. Trials where the task cue was the same as on the preceding trial are referred to as stay trials, while trials where the task differed from the prior trial are called switch trials. A significance level of p < 0.05 was adopted for all analyses (unless otherwise specified).

### Behavioural analyses

In the study phase, the proportions of correct left/right judgments were close to ceiling, with no significant difference between the two valence categories in terms of accuracy or reaction time (RT) [ts(31) <0.90, ps > 0.37]. This indicates that participants were paying attention in the study phase and correctly encoding items. Next it was determined whether participants could do the task i.e. whether performance was better than chance levels. Collapsing across valence, for the episodic task, the likelihood of a correct old response to an old word, irrespective of the accuracy of location judgements, was greater than the likelihood of an old response to a new word for switch and stay trials [means of 0.88 vs 0.06 for switch and 0.90 vs 0.06 for stay, t(31)s > 45.81, p < 0.001]. For both switch and stay trials the mean accuracy of location judgements were reliably above chance [both t(31)s > 40.16, p < 0.001].

Response accuracy for the episodic and perceptual tasks in the test phase are shown in Table 1. A within-subjects ANOVA was conducted to determine if valence or trial type had an effect on participants’ performance. This was completed on accuracy data (hits) with factors of valence (negative, neutral), task (episodic, perceptual), and trial type (switch, stay). There was a main effect of task [F(1,31) = 38.60, p < 0.001], reflecting a higher level of accuracy in the perceptual task relative to the episodic task across switch and stay trials. There were no main effects or interactions involving valence or trial type. Thus, no switch costs were observed in accuracy data.

**Table 1:** Response accuracy for the episodic and perceptual tasks on switch and stay trials separated by valence (standard deviations in parentheses).

	Negative		Neutral	
	Switch	Stay	Switch	Stay
Source Hits	0.76 (0.10)	0.77 (0.10)	0.76 (0.14)	0.78 (0.14)
Correct Rejections	0.94 (0.05)	0.94 (0.07)	0.95 (0.06)	0.95 (0.06)
Perceptual Hits	0.88 (0.11)	0.88 (0.09)	0.89 (0.10)	0.90 (0.09)

**Figure 2:**
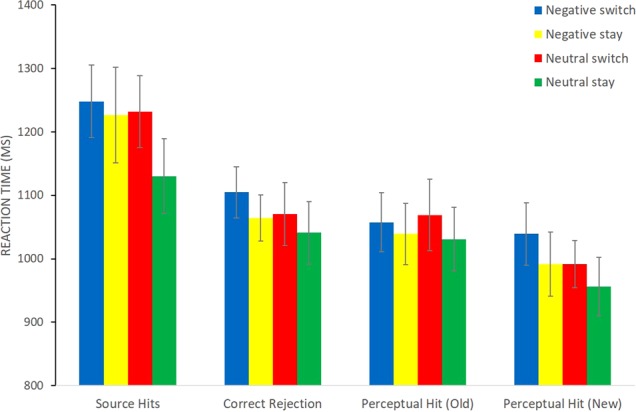
Mean RTs of correct responses for each valence (negative/neutral), task (episodic/perceptual), trial type (switch/stay), and picture status (old/new). Error bars represent within-subject confidence intervals^52^.

Similarly, a within-subjects ANOVA was conducted on RT data for correct responses to determine if valence or trial type affected the speed that participants completed the tasks. This had factors of valence, task, trial type, and picture status (old, new). There was a main effect of trial type [F(1,31) = 19.01, p < 0.001], indicating that overall participants were slower on switch trials compared to stay i.e. a switch cost was found in RT data. In addition, there were also main effects of task [F(1,31) = 44.80, p < 0.001] and picture status [F(1,31) = 41.83, p < 0.001], reflecting faster responses in the perceptual task compared to episodic and also to new pictures relative to old. There were also two interactions: task × picture status [F(1,31) = 13.70, p = 0.001], and valence × task × picture status [F(1,31) = 5.82, p = 0.022]; reflecting the RT difference between tasks was larger for old than new pictures, and this pattern was more pronounced for negative relative to neutral pictures. The RT data are presented in Fig. 2.

### ERP analyses

All analyses included the Greenhouse-Geisser correction^53^ for nonsphericity when necessary. Epsilon-corrected degrees of freedom are reported. The mean number of trials and ranges for each condition are included in a supplementary table.

In previous ERP studies examining memory retrieval of IAPS pictures the neural index of recollection has been measured between 500–700 ms^54,55^ and so the same time window was adopted in this study. As our focus was on recollection and the left-parietal ERP old/new effect we also constrained our analyses a priori to the sites that this effect is usually maximal i.e. left-parietal (P1, P3, P5 and P7). Only reliable effects involving picture status i.e. old/new effects, are highlighted below.

### Perceptual task

**Figure 3:**
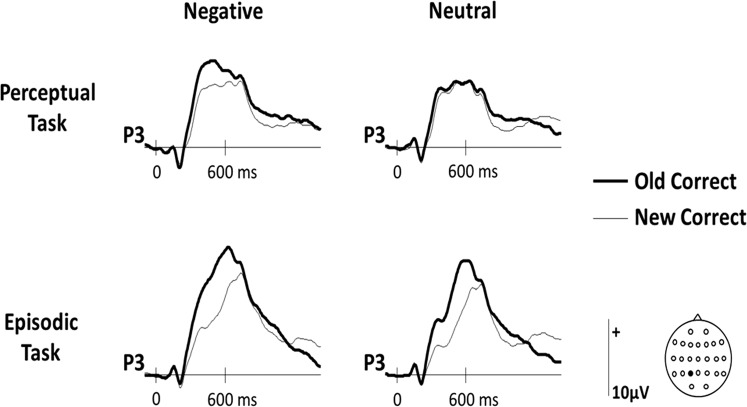
Grand-average ERP waveforms from a left posterior superior electrode site (P3; indicated by the head diagram at the bottom right) for the perceptual and episodic tasks collapsed across trial type and split by valence. For the perceptual task the waveforms are for when the participant gave correct responses to the current screen locations of old and new pictures and for the episodic task they represent when the locations of old pictures were retrieved correctly and new pictures were identified correctly.

To address the main aim of this study: to determine the effects of emotional stimuli on the ability to curtail remembering when it is not required for the current task, an ANOVA was conducted on ERPs for correct items from the perceptual task. At issue is whether left-parietal ERP old/new effects will be found in the perceptual task, where there is no requirement to retrieve episodic information, and whether it will differ by valence. The ANOVA included the factors: valence (negative, neutral), trial type (switch, stay), picture status (old, new) and site (P1, P3, P5, P7). This revealed an interaction between valence and picture status, F(1, 31) = 4.33, p < 0.05 and also an interaction between picture status and site, F(2.4, 75.6) = 7.08, p = 0.001. These interactions were followed up by examining old/new effects and valence at each electrode site (Bonferroni corrected for the 4 sites, p = 0.0125). There were no significant effects of picture status at P1, P5 or P7 (even without a Bonferroni correction, p > 0.05). At the P3 electrode there was an interaction between valence and picture status, F(1, 31) = 10.77, p = 0.003. When this was examined separately by valence a significant old/new effect was found for the negative stimuli, F(1, 31) = 11.38, p = 0.002, but no significant effects of picture status were found for neutral stimuli. There was no significant main effect or interaction involving trial type, so waveforms are displayed collapsed across switch and stay trials in Fig. 3. The mean amplitude difference between responses to old pictures and responses to new pictures was 1.60 µV for negative stimuli and 0.05 µV for neutral stimuli – Cohen’s d_z_ = 0.60 and 0.02 respectively.

### Episodic task

To demonstrate that ERP left-parietal old/new effects are observed when participants are asked to retrieve episodic information an ANOVA was conducted on data from the episodic task. This ANOVA contained the same factors as described above for the perceptual task, but for picture status old items were source hits and new items were correct rejections. There was an interaction between picture status and site, F(2.3, 71.9) = 8.05, p < 0.001 and a main effect of picture status, F(1, 31) = 56.84, p < 0.001. The interaction was followed up by examining old/new effects at each electrode site (P1, P3, P5, P7). Old/new effects were present at all electrode sites, Fs(1, 31) = 37.82–67.70, ps <0.001, and were numerically largest at mid-lateral sites. Thus, left-parietal old/new effects are evident in the episodic task, as anticipated, but these do not differ according to valence of stimuli. Waveforms can be seen in Fig. 3.

### Rumination

Correlations were conducted to determine whether there was any relationship between trait rumination and the extent to which participants continued to recover memory information when it was not required. To quantify the extent of unwanted recollection the magnitude of the left-parietal old/new ERP effect at P3 in the perceptual task was examined - collapsed across switch and stay trials due to the lack of any significant main effects or interactions involving this factor. As rumination scores were not normally distributed Spearman’s correlations were completed. A significant relationship was found between the magnitude of the left-parietal ERP old/new effect to neutral stimuli and rumination scores [ρ(30) = 0.39, p = 0.01], but no significant association was present for negative stimuli [ρ(30) = −0.29, p > 0.05]. The Ruminative Responses Scale contains items which overlap with phenomenological experiences of depression, so to unconfound these two constructs researchers have excluded these items and found that two factors emerge for the remaining items: reflection and brooding^47^. In a further exploratory analysis we scored participant’s questionnaires according to these two factors and examined the relationship with the magnitude of the left-parietal old/new effect to neutral stimuli. A significant relationship was only found with the brooding factor [ρ(30) = 0.46, p = 0.004] and not reflection [ρ(30) = 0.25, p > 0.05]. Note that there were no significant correlations between the magnitude of the left-parietal ERP old/new effect to negative stimuli and these two factors (ρs = −0.12 and −0.14).

## Discussion

The aim of this study was to examine whether people can exert control over retrieval and prevent this from occurring when it is not required. Importantly the task we utilised to examine this did not explicitly ask participants to try to suppress information from memory, unlike the TNT paradigm. Instead, the demands of the task changed so that sometimes the retrieval of episodic information was required whereas on other trials it was not, and participants needed to manage these different demands. For correct responses in the perceptual task the neural index of recollection was present for negative stimuli on both switch and stay trials but not for neutral stimuli, supporting our hypothesis that participants would be less able to curtail remembering when the stimuli were negative. Furthermore, in an exploratory analysis there was a significant positive correlation between the magnitude of the left-parietal ERP old/new effect in the perceptual task (collapsed across switch and stay trials) for neutral stimuli and rumination, but not for negative stimuli. Thus, participants who had higher scores on the rumination scale showed more evidence of continuing to recall episodic information when it was not required, but this was only to neutral stimuli. In a further exploratory analysis a positive association was found between the magnitude of the left-parietal ERP old/new effect in the perceptual task to neutral stimuli and the construct of brooding but not reflection. This indicates that those individuals who subjectively report that they passively focus on their negative experiences are more susceptible to intrusions from memory when they are presented with neutral stimuli.

As outlined in the Introduction there have been two opposing lines of thought about participant’s ability to exert control over emotional material compared to neutral. On the one hand it has been suggested that greater cognitive control can be exerted over negative stimuli compared to neutral, which will lead to better memory suppression for negative stimuli^11–13^. On the other hand, negative stimuli may be more difficult to exert control over because they are thought to capture attention more easily^56^, which enhances encoding^57^ and retrieval^20^. In line with the latter view, many studies have found that emotional memories are retained better than neutral memories (reviewed by^19,20^). The electrophysiological data from the current study also support this view: overall participants were not able to control memory when presented with negative stimuli and continued to retrieve even though it was not required for the task at hand, but they could do this for neutral stimuli.

There are a couple of other studies which have utilised electrophysiological data to determine the extent of remembering of emotional stimuli within the context of the T/NT paradigm^25,27^. In these studies the left-parietal ERP old/new effect is examined in the second stage of the task where participants see an item and are instructed to either retrieve the associate (think condition) or not (no-think condition). This is a clear difference to the task used in the current study where electrophysiological data are collected in the test phase. Chen *et al*.^25^ tested healthy volunteers using faces which were associated with negative or neutral pictures. Behaviourally, negative pictures from the no-think condition were recalled significantly more than neutral pictures and both valences had lower recall than the baseline items. Electrophysiologically, in the no-think condition there was a significantly larger left-parietal old/new effect, at C3 and P3, to negative stimuli in comparison to neutral. The magnitude of this effect did not differ between the different valence categories in the think condition. Thus, this study offers some interesting parallels with the current study: negative memories are harder to suppress than neutral ones but when participants are explicitly asked to retrieve from memory there is no difference between the valence categories.

However, a difference in the left-parietal ERP old/new effect in the no-think condition between negative and neutral valence stimuli is not always present. Zhang *et al*.^27^ conducted a TNT study with participants split into high and low depression groups. Behaviourally, when collapsing across groups they found that more negative no-think items were recalled in the final test compared to neutral and this pattern of result was more extreme in the depressed group. However, no difference in the left-parietal ERP old/new effect was found between the negative and neutral no-think items in either group. Thus, there is a disconnect in the behavioural and electrophysiology data in this study: the behavioural data indicate that participants find negative content harder to suppress but there is no evidence for this when looking at the left-parietal ERP old/new effect. It is important to note that these different types of data come from different stages of the experiment: behaviour from the final test and ERPs from the second suppression stage. Using the TNT paradigm it is known that forgetting increases with the number of repetitions of the no-think items^7^. It is possible that when no-think items are averaged across all repetitions a left-parietal old-new effect is found because it was present to earlier repetitions of the item and attenuated or abolished to later items. This could explain why a different pattern of results might be observed between the electrophysiological and behavioural data i.e. whether participant’s demonstrate suppression or not. However, it does not explain the discrepancy in the electrophysiological data between this study and Chan *et al*.’s^25^. Moreover, it is hard to understand why these studies found such different results, as the design of the TNT paradigm is almost identical between them.

Our findings related to rumination are generally consistent with the results from other studies^42,43,58^, but it is unusual that we only found relationships with neutral stimuli and not negative. A critical question is whether ruminators will show a general memory control deficit or a specific difficulty with controlling emotionally negative stimuli. Dieler *et al*.^59^ used the TNT paradigm with pairs of neutral faces which were associated with either negative or neutral target pictures. They found that those participants high in the brooding aspect of rumination (the same dimension of rumination that we found) showed reduced suppression to the no-think items, but this was only for negative targets. This finding contrasts with other studies which have examined cognitive control and found that rumination related deficits are unaffected by valence in non-depressed populations^60^. Indeed, some theorists have suggested that a more general problem with disengaging attention from extraneous information is what predisposes an individual towards rumination initially^41^. In the study by Fawcett *et al*.^42^ they used neutral stimuli and found a relationship between high rumination and an impaired ability to suppress memories. They also suggest a number of reasons for the difference with the Dieler *et al*.^59^ study, including: whether participants were screened for psychiatric disorders, differences in the intertrial interval in the TNT stage and the specific instructions used. If ruminators have a more general memory control deficit it would be anticipated that relationships should have been found with both neutral and negative stimuli in the current study. This was not the case.

It is puzzling why we found that rumination, and brooding in particular, was associated with a failure to prevent retrieval of task-extraneous material when neutral stimuli were used, but there was no relationship when examining the negative stimuli. Examining the data in more detail we found that the vast majority of participants (25/32) exhibited left-parietal old/new effects to negative stimuli, and these were significant at the group level. In contrast, we did not find an overall significant left-parietal old/new effect to neutral stimuli in the perceptual task, this was because half of the participants demonstrated an effect whereas the other half did not. It is instructive here to consider the process of recollection and what exactly the left-parietal ERP old/new effect is measuring. There has been extensive debate about which model might most accurately describe the process of recollection^61,62^. There are now a number of studies supporting the some-or-none model e.g.^63^, which proposes that recollection is thresholded i.e. absent or present, and when successful can vary. Indeed, ERP studies examining the left-parietal old/new effect have shown that this index varies with the precision of the information recovered^64^ and the amount of contextual details remembered^65,66^. Therefore, the absence of a left-parietal ERP old/new effect would indicate no recollection. However, the presence of an effect, and its magnitude, could reflect not just that recollection has occurred but also the amount/precision of information recovered i.e. the measure conflates these two aspects.

Turning to this study, the majority of participants demonstrated a left-parietal effect for negative stimuli in the perceptual task, so recollected information. Moreover, it is known from previous research that unpleasant stimuli are associated with the recovery of more contextual details^16,18,21,22^. Thus, the magnitude of the left-parietal effect that is observed in this condition is likely to largely be measuring the amount of contextual information participants recover. In contrast, when neutral pictures were used in the perceptual task half of the participants did not display a left-parietal ERP effect, and so did not recollect. The rest of the participants did exhibit this neural effect, but this may be associated with fewer contextual details than the negative stimuli^16,18,21,22^. Supporting this latter assertion, in this study it was found that for those who demonstrated a left-parietal old/new effect the magnitude was larger in the negative condition compared to neutral (2.51 vs 2.02 µV). Consequently, in the neutral stimuli condition the left-parietal index, for the most part, is likely to reflect the presence or absence of recollection and is less likely to be affected by the amount of information retrieved compared to negative stimuli. Thus, if rumination is associated with a failure to stop recollection (rather than how much contextual information is recollected) then a relationship with the neutral stimuli would more likely be found. This is because in this condition the left-parietal index is a purer measure of the presence of recollection, whereas it is more conflated in the negative condition with the amount of recollected information. This might explain the pattern of correlational results obtained but why would individuals high in rumination, and brooding, be unable to prevent retrieval of task-irrelevant information when neutral stimuli are used? One possibility is that those high in this trait may have a tendency to interpret even ostensibly neutral pictures in terms of a negativity bias. These are post-hoc explanations for these results and clearly more research is needed to determine if they are correct.

The absence of a left-parietal ERP old/new effect to neutral stimuli in the perceptual task, on either switch or stay trials in the current study, would suggest that participants were able to prevent memory retrieval of these items. This finding contrasts with our previous study^38^ where we found a left-parietal ERP old/new effect on the switch trial of the non-episodic task but not the stay trial. There are a few differences between these studies. The first is that in the current study colour photographs were used whereas words were the stimuli in the previous study. Secondly, in this study there was double the number of study-test cycles compared to Evans *et al*.^38^. Finally, the other difference between the studies is that even though the perceptual task was the same between these two studies (judge current screen location of the item: above, at or below fixation) participants found it more difficult in the current study, as evidenced through longer RTs and lower levels of accuracy. These factors would need to be examined in further detail to determine how they affect memory control ability.

The results from the current study also have relevance to the task switching literature. A RT switch cost was found which is consistent with the broader task switching literature, where participants are typically quicker when completing subsequent trials of the same type^45^. Moreover, RT switch costs have also been found in other papers which have used a task switching design and tested memory, either by examining recall of contextual information i.e. recollection^5,38,46,67^ or with an old/new judgement^3,67^. No switch cost was found in the accuracy data, which again is quite typical of these task designs, although see^49^ for a paper which did find accuracy switch costs and a detailed discussion of this. Broadly, there are two main accounts for the switch cost, see reviews by^68,69^. Important to both is the concept of task set, which is a collection of cognitive processes required to perform a task. According to the task set reconfiguration explanation^70,71^ the switch cost reflects the time it takes on the first trial of a new task to engage control processes to reconfigure the system to complete a new task; which could include shifting attention or criteria, retrieving what to do and how to do it, as well as inhibited the previous task set and activating the new one. An alternative view is called task set inertia^72^, which proposes that when participants switch tasks they still have a tendency to perform the previous task which interferes with their ability to perform the current task, resulting in a switch cost.

The RT switch cost found in the current study cannot disentangle these two accounts, but the left-parietal ERP old/new effect may shed light on the underlying cognitive processes. The ERP findings provide evidence to support the task set inertia account as when participants were completing the perceptual task, where there was no requirement to recover episodic information, the neural index of recollection was present to negative stimuli. However, this index was present on both the switch and the stay trials which suggests that the RT switch cost in this study is not solely a function of the interference caused by continuing to complete the previous task as this would be expected to reduce or be resolved on the second trial. Furthermore, there was no neural evidence for task set inertia when neutral stimuli were examined but a RT switch cost was found. This might support the task set reconfiguration account, as perhaps participants were inhibiting the previous task set, which resulted in no evidence for a left-parietal ERP old/new effect at the group level to neutral stimuli. It seems to be the case that rather than the switch cost being a result of task-set inertia or task set reconfiguration this depends upon the specific experimental circumstances. The paradigm used in the current study might offer a useful means of determining the parameters under which each of them operates.

Traditionally research into task switching and long-term memory has tended to follow separate paths but there could be areas of overlap. In task switching, the concept of a task set describes the cognitive processes needed for individuals to make different responses according to the goal of the specific task they are completing. For example, in a typical task switching experiment participants have to switch between completing tasks with different demands on the same stimulus, such as classifying a number as odd/even or high/low. A parallel concept in memory research is the notion of memory states, such as retrieval mode. Retrieval mode is a state which people enter when there is a requirement to retrieve episodic information^1^, for example, details of your last birthday. In the current study this state was examined by asking participants to switch between retrieving episodic information from the study phase, where retrieval mode would be engaged, versus a perceptual judgement, which does not require it. Just like in conventional task switching designs the stimuli afforded participants the opportunity to complete different tasks on the same stimuli. Thus, the concept of retrieval mode and the related memory state of retrieval orientation^73^ (the retrieval of task-relevant episodic information e.g. remembering who came to your birthday party versus what food was served) seem to share features with task sets^74^. This has been examined in a meta-analysis^74^ and it was found that there is substantial overlap in the neural mechanisms implicated in the control of task sets and memory states. Given the conceptual and methodological overlap between these two fields it would seem useful to investigate this in more detail to determine their similarities and differences and what this might mean theoretically.

In conclusion, we have developed a novel emotional paradigm which does not have an explicit instruction to suppress memory content but instead examines participant’s ability to disengage episodic memory processing when it is not required for the task at hand, and so is complementary to the frequently used TNT task. We found that participants can control memories that are unwanted for the completion of the current goal when presented with neutral stimuli. However, it is difficult to stop memory intrusions when presented with negative stimuli. The trait of rumination, and in particular brooding, was found to be correlated with individual differences in the ability to prevent unwanted memory retrieval when neutral pictures are used. Thus, there are interactions between valence and rumination which affect people’s ability to control their memories, which should be explored in more detail as they may have important implications for general wellbeing and clinical disorders.

## Supplementary information

Supplementary Information

## Data Availability

Are available to researchers on reasonable request by contacting the corresponding author.
