# Supplementary material for: Neural evidence that disengaging memory retrieval is modulated by stimulus valence and rumination
Source: Sci Rep. 2020 May 5;10:7548. doi: 10.1038/s41598-020-64404-7 (PMC7200702; doi:10.1038/s41598-020-64404-7)
Supplement: Supplementary file 1 — Supplementary Information [file 41598_2020_64404_MOESM1_ESM.docx]

Neural evidence that disengaging memory retrieval is modulated by stimulus valence and rumination.

Jiangyi Xia^1^ & Lisa H. Evans^2^*

^1^ Centre for Mind and Brain, 1 Shields Avenue, UC Davis, California, 95616.

^2^ Cardiff University Brain Research Imaging Centre (CUBRIC), School of Psychology, Cardiff University, Cardiff, CF24 4HQ, Wales, UK.

* Corresponding Author: [EvansLH@cardiff.ac.uk](mailto:EvansLH@cardiff.ac.uk)

Supplementary Table

Means and ranges of artefact-free trials included in ERP analyses for each condition.

|  | *Mean* | *Range* |
| --- | --- | --- |
| *Negative pictures* |  |  |
| Source hit switch | 21 | 16-28 |
| Source hit stay | 21 | 16-29 |
| Correct rejection switch | 26 | 17-30 |
| Correct rejection stay | 26 | 20-30 |
| Perceptual hit switch (old) | 25 | 16-30 |
| Perceptual hit stay (old) | 25 | 16-29 |
| Perceptual hit switch (new) | 24 | 17-30 |
| Perceptual hit stay (new) | 24 | 18-30 |
| *Neutral pictures* |  |  |
| Source hit switch | 21 | 16-28 |
| Source hit stay | 21 | 16-29 |
| Correct rejection switch | 26 | 20-30 |
| Correct rejection stay | 27 | 20-30 |
| Perceptual hit switch (old) | 25 | 16-30 |
| Perceptual hit stay (old) | 25 | 16-30 |
| Perceptual hit switch (new) | 24 | 16-29 |
| Perceptual hit stay (new) | 25 | 16-30 |
